# Maternal mortality as a Millennium Development Goal of the United Nations: a systematic assessment and analysis of available data in threshold countries using Indonesia as example

**DOI:** 10.7189/jogh.07.010406

**Published:** 2017-06

**Authors:** Evelyn Reinke, Supriyatiningsih, Jörg Haier

**Affiliations:** 1 University Hospital Münster, Münster, Germany; 2 Medical Services of Asri Medical Center, Universitas Muhammadiyah Yogyakarta, Yogyakarta, Indonesia; 3 Nordakademie University of Applied Sciences, Hamburg/Elmshorn, Germany

## Abstract

**Background:**

In 2015 the proposed period ended for achieving the Millennium Development Goals (MDG) of the United Nations targeting to lower maternal mortality worldwide by ~ 75%. 99% of these cases appear in developing and threshold countries; but reports mostly rely on incomplete or unrepresentative data. Using Indonesia as example, currently available data sets for maternal mortality were systematically reviewed.

**Methods:**

Besides analysis of international and national data resources, a systematic review was carried out according to Cochrane methodology to identify all data and assessments regarding maternal mortality.

**Results:**

Overall, primary data on maternal mortality differed significantly and were hardly comparable. For 1990 results varied between 253/100 000 and 446/100 000. In 2013 data appeared more conclusive (140–199/100 000). An annual reduction rate (ARR) of –2.8% can be calculated.

**Conclusion:**

Reported data quality of maternal mortality in Indonesia is very limited regarding comprehensive availability and methodology. This limitation appears to be of general importance for the targeted countries of the MDG. Primary data are rare, not uniformly obtained and not evaluated by comparable methods resulting in very limited comparability. Continuous small data set registration should have high priority for analysis of maternal health activities.

In 2000 governments from 189 countries agreed on the Millennium Explanation (Millennium Development Goals – MDG) at one of the largest summits of the United Nations (UN). Eight global development objectives were defined and the world community aimed to achieve these goals until 2015. In addition to the fight against poverty and starvation, for equalization of nations and individuals the fifth MDG contains the aim to improve the health care of mothers. The maternal mortality has been considered as an important indicator for the health of the population and the economic as well as social development. Based on this prioritization the targeted worldwide reduction of maternal mortality rates was targeted to be reduced by about 75% [1].

Major problems related to high maternal mortality currently occur in Africa, but also in Asia, in particular in South–East and South Asia. However, comparison between countries and over time requires sufficient data quality based on standardized definitions and data availability with satisfying representability. Here, we systematically examined available data quality of maternal mortality in Indonesia as one of the largest countries worldwide with high impact on the achievements of the Millennium Goals. The aim of this analysis was the evaluation, critical review and summary of all available primary data about maternal mortality in Indonesia during the MDG time period. Specifically, we reviewed data resources and their acquisition before the Millennium Explanation and at the end of the proposed timeframe for their achievement. The critical review focused especially toward coverage of available data for the whole population of the country, reliability and potential biases of data acquisition. Comparable to other countries, such as Cambodia [2] and India [3], different record sets and a large gap of primary data were identified for Indonesia.

## Definition of maternal mortality

For maternal mortality, the World Health Organization (WHO) defines a maternal death as the death of a woman during pregnancy or within 42 days after its end regardless of duration or place of the pregnancy. It is distinguished between direct and indirect cause of death. A direct death is caused by complications directly related to the pregnancy, childbirth and puerperium including interventions, omissions, insufficient treatments or their combinations. An indirect death originates from preexisting illnesses which have worsened by physiological changes of the pregnancy [4]. A systematic WHO study investigating the causes of the maternal mortality worldwide raised that more than one quarter of these deaths are due to indirect causes. The systematic analysis showed that main direct causes of death (overall 73%) were bleeding (27%), followed by hypertension (14%) and sepsis (11%) [5].

## Methods to measure maternal mortality

Different methods to determine the risk of a motherly death have been used so far: maternal mortality ratio (MMR, for distinction also MMRatio) determines the number of the mothers’ deaths per 100 000 live births per time period. Maternal mortality rate (for differentiation MMRate) defines the number of the maternal mortality in a certain period per 100 000 women at reproductive ages. The lifetime risk considers the likely hood to become pregnant and the likelihood of mothers’ mortality at the reproductive ages (WHO, 2011) [4]. As alternative indicator for maternal mortality the percentage of pregnancy related deaths divided by all deaths with women at the age of from 15 to 49 years (PMDF) has also been implemented. Overall, to reliably obtain indicators for maternal mortality remains a difficult challenge mainly due to context–specific interrelations of various required information [6]. The industrial nations often dispose efficient registration systems enabling them to raise sufficient reporting. However, in the developing and threshold countries these data are often not documented in a reliable and complete manner. Due to the lack of real life data information are often used based on (randomly) selected subpopulations. For example, this includes specific questions within the scope of national censuses, budgetary questionnaires or more specific family interviews [7].

## METHODS

### Data sources

A comprehensive analysis of known international health care reporting resources combined with a systematic review according to the Cochrane method was carried out to identify the available primary data and studies that provide maternal mortality information for Indonesia. The elevated data, acquisition techniques, calculation methods and study designs were validated and compared between the various reports and for the proposed MDG period.

As initial step international and national health care reporting resources were searched for available data about mothers’ mortality including:

- WHO
- United Nations Population Fund
- Worldbank
- Badan Pusat Statistik Indonesia
- The Organization for Economic Co–operation and Development (OECD)

To verify the results found at the international reporting platforms and to identify other data about the MMR a systematic review was subsequently carried out using the search criteria: “maternal mortality” and “Indonesia”. All obtained published paper were evaluated according to the criteria of the Cochrane Collaboration for meta–analyses [8]. Although this review is not based on clinical trials the principles and structure of this method have been adapted accordingly.

### Targets for evaluation

The targeted questions or aims of the primary reports refer to the central issue of this critical review. It was carefully looked for the different types of indicators for maternal mortality and for the types of data acquisition. This was of high importance since Indonesia (as most other threshold countries) does not provide a full–coverage and nationwide registration system. Specifically, the following criteria were used for upfront comparison:

- Targeted indicator
- Targeted population (if applicable selection criteria)
- Measured data, secondary resources (eg, family reporting) or estimations.

According to the scope of this review and in order to avoid secondary referencing effects primary reports were included in this evaluation exclusively. Data acquisition performance was evaluated and compared regarding the used baseline data, elevation methods, study design and kind of reporting. An assessment of the statistical calculation models was not done, but descriptions are given for completeness of the analysis.

### Inclusion and exclusion criteria

For this review full–paper publications and studies in English–speaking journals were primarily used. These publications were checked if they provide primary information and/or estimates about the Indonesian maternal mortality. Reports only based on references were expelled from the evaluation but will be included in the discussion. If the identified references did not contain MMR data for Indonesia and/or data were only referenced from other publications those were excluded.

### Literature search

A systematic search for Medical Subject Headings (MeSH)”Maternal Mortality” and “Indonesia” was carried out. The search was limited to the period of the years 2000 to 2014. Secondary literature was included if those references fulfilled the inclusion criteria. In addition, resources in Indonesia (especially governmental) supplemented the evaluation of the available data.

### Data extraction

All reports that were suitable for detailed review were analyzed regarding the following information.

- Type of data acquisition
- Whole coverage versus selected population
- Direct or indirect measurement
- Methods of data assessment and aggregation
- Inclusion of cofactors

### Validation and comparability

To evaluate the reported data, their inter–observer and longitudinal comparability for all selected publications the following aspects were considered.

- Representability of data
- Potential selection bias
- Reliability of obtained data
- Reproducibility of maternal mortality indicators

### Search for publications and reports

With respect to the review of literature, DIMDI and PubMed were searched, with DIMDI accessing approx. 30 international databases, such as MEDLINE, EMBASE, SciSearch and Cochrane Library. In doing so, a total of 262 publications were identified, 56 relevant publications of which found in PubMed. Subsequently, titles and abstracts were read, while relevant literature was further investigated by reading and analyzing the full text. Numerous publications could be found in both DIMDI and PubMed, and identical writings were identified and collated. Apart from methodically searching the above–mentioned databases, a non–systematic search was carried out, reviewing the results of the methodical literature search by snowball procedure. In doing so, another nine publications were determined. Accordingly, a total of 327 abstracts were identified. Five publications were considered after the selection.

## RESULTS

As shown in **Table 1** it is evident that different data and methods for maternal mortality calculations in various primary reports were applied. This resulted in variations of the reported MMR for 1990: 253–446/100 000, for 2000: 265–420/100 000 and for 2010: 165–346/100 000. In addition, the “Indonesian Demographic and Health Surveys” (IDHS) in 2012 even reported 359/100 000 [14]. Subsequently, the identified primary data reports will be described.

**Table 1:** Reported MMR data for Indonesia since 1990

Author	Title	1990	1995	1997	2000	2002 and 2003	2005	2007	2008	2010	2011	2012	2013
Statistics Indonesia; National Population and Family Planning Board; Ministry of Health	Demographic and Health Surveys (Indonesia), 1987, 1991, 1994, 1997, 2002–2003, 2007 and 2012			390		307		228				359	
MMEIG (WHO, UNICEF, UNFPA and The World Bank)	Trends in Maternal Mortality, 1990–2008,1990–2010, 1990–2013 (data for 2013)	430	360		310		250						190
United Nations	The Millennium Development Goals Report, 2008, 2009, 2010, 2011, 2012, 2013, 2014 (data for 2014)	446	326	299	265	241/231	212	190	181	165	156	148	140
Central Bureau of Statistics (BPS) of Indonesia	Sensus Penduduk, 2010									346			
Harvard Center for Population and Development Studies (Hill et al.); 2007 [9]	Estimates of maternal mortality worldwide between 1990 and 2005: an assessment of available data				420								
Institut für Health Metrics and Evaluation (Hogan et al.); 2010 [10]	Maternal mortality for 181 countries, 1980—2008: a systematic analysis of progress toward Millennium Development Goal 5	253			290				229				
Institut für Health Metrics and Evaluation (Lozano et al.) 2011 [11]	Progress toward Millennium Development Goals 4 and 5 on maternal and child mortality: an updated systematic analysis	404			333						248		
Institut für Health Metrics and Evaluation (Kassebaum, et al.); 2014 [12]	Global, regional, and national levels and causes of maternal mortality during 1990–2013: a systematic analysis for the Global Burden of Disease Study 2013	368				262							199
Ronsmans et al.; 2008 [13]	Professional assistance during birth and maternal mortality in two Indonesian districts						435						

At national level, household IDHS survey data could be identified. Data acquisition of maternal mortality was done as “sister’s survey”. To receive different records on the maternal mortality, siblings of surviving relatives were interviewed using a detailed questionnaire about surviving relatives of all living born children of the questioned mother. The MMR in 1997 IDHS was 390/100 000. Unpublished analysis of data from the IDHS show a slight decline to 334/100 000 births for the period 1993–1997. Subsequent surveys showed further declined MMR until 2007 (228/100 000), but a subsequent increase in 2012 to 359/100 000 [7]. According to the authors, IDHS suffer from selection bias and high confidence intervals of the survey data. However, the general increased rate of reported adult female mortality in Indonesia appears to be consistent with this MMR increase. One important selection bias should be considered due to the fact that until 2007 only married women were questioned, but in 2012 unmarried women were also interviewed. In addition, in 2007 siblings of both genders were included whereas in 2012 only female siblings were interviewed. According to these primary data from the period 1993–1997 to 2012 the annual rate of reduction (ARR) would only be 0.4%.

At supranational level two reports were raised by the United Nations. The “Trends in Maternal Mortality” reports are a number of analyses published by the WHO, UNICEF, UNFPA, the World Bank and United Nations [15]. Headed by the Maternal Mortality Estimation Group (MMEIG) maternal mortality estimates were developed [16]. In 2008 and 2010 included countries have been classified into groups A – C according to the availability and quality of maternal mortality data to evaluate estimation methods of MMR for each country. Countries which had data from civil registration systems were classified as Group A (countries with no available national–level data on maternal mortality) and B (countries that lack complete registration systems but for which other nationally representative data are available for measuring maternal mortality) whereas group C consists of countries for which multi–level regression models were applied for lack or incomplete registration data. This model uses socioeconomic information including fertility, birth attendants and Gross Domestic Product (GDP) as well as available national maternal mortality data. Nearly half of all countries including Indonesia belong to group B. Therefore, in this critical overview the authors consider Indonesia as example of the majority of threshold countries. Only data from IDHS until 1994, from 2002 to 2003, 2007 and 2012 were used. This is in line with the data limitations, modeling assumptions and quit large uncertainty intervals around global maternal mortality estimates that have been recognized by MMEIG [17].

The MDG Reports are based on a set of comprehensive official statistics compiled by the Inter–Agency and Expert Group (IAEG) on MDG indicators led by the Statistics Division of the Department of Economic and Social Affairs [18]. The reports use population adjusted means of official data provided by governments, international agencies and, if available, Demographic and Health Surveys. Transparency of data resources is ensured by IAEG (http://mdgs.un.org), but data quality relies on national publication policies. For South East Asia apart from Indonesia, the following countries are summarized in Southeast Asia: Brunei Darussalam, Cambodia, Lao People’s Dem Republic, Malaysia, Myanmar, Philippines, Singapore, Thailand, Timor–Leste. For Indonesia the web–based publication refers to data and rather methodology of the WHO’s report “Trends in maternal mortality” [IAEG]. The UN’s ARR being at –4.9% between 1990 and 2013 is higher than the decline obtained by MMEIG (–3.5%) during the same period.

“Sensus Penduduk” (SP) is a national census and grasps socioeconomic and demographic information like age, religion, impediment, ethnic origin, language, migration, education, marital status, employment, fecundity, mortality and residential facilities of the population. SP results in 2010 are still incompletely published, originally reporting MMR of 259/100 000 live births, after recalculation being 346 per 100 000 live births [19,20].

Using Cochrane search strategy five additional publications could be found (**Table 2**). However, these publications do not contain primary data and include multinational summaries. For Indonesia different types of data were considered reaching from siblings’ surveys, case-control studies and estimations. Different types of calculations were used and indicators were also not comparable throughout the reports.

**Table 2:** Results of Cochrane–based data evaluation

Author	Title	Number of countries	Number of data	Data used from Indonesia	Period	Method	HIV	Statistical calculate model
Hill et al.; 2007 [9]	Estimates of maternal mortality worldwide between 1990 and 2005: an assessment of available data	125	858	63 sisterhood studies for group C	1985–2005	PMDF	yes	random–effects time–series regression model for group A+B; for the groups C until H a model of PM with 2 coefficients
Hogan et al.; 2010 [10]	Maternal mortality for 181 countries, 1980—2008: a systematic analysis of progress toward Millennium Development Goal 5	181	2651	2 verbal autopsies, 5 sibling histories, 4 WHO estimates	1980–2008	PMDF	yes	Linear model an spatial–temporal local regression with 6 covariates
Lozano et al.; 2011 [11]	Progress toward Millennium Development Goals 4 and 5 on maternal and child mortality: an updated systematic analysis	187	3793	9 verbal autopsies, 8 survey/census, 60 sibling histories	1980–2011	MMRatio	yes	Linear model an spatial–temporal local regression with 11 covariates
Kassebaum, et al.; 2014 [12]	Global, regional, and national levels and causes of maternal mortality during 1990–2013: a systematic analysis for the Global Burden of Disease Study 2013	188	7065	3 verbal autopsies, 6 IDHS, 2 surveys/census	1980–2013	MMRatio	yes	Codem–Model with 9 covariates
Ronsmans et al.; 2008 [13]	Professional assistance during birth and maternal mortality in two Indonesian districts			Sample: 458 cases, 1234 controls	01/2004 – 12/2005	MMRatio	no	Capture–recapture method

Hill et al. utilized a number of different data records to generate an estimate for 125 countries, and international sources (local data collections) as well as WHO, UNICEF, UNFPA information and further national sources were considered. In 2005, there were an estimated 402 global maternal deaths in 100 000 live births (confidence interval CI 216–654). They applied a concept similar to MMEG’s: According to availability and quality of the data (civil registration or complete registration of deaths, direct sisterhood or reproductive age mortality surveys, estimates based on sample registration, population census or empirical data), countries were classified into eight groups (A to H). In groups C, F and H, maternal mortality was assessed according to the PDMF. The acquisition period was 1985 to 2005. Indonesia as well as another 27 countries with incomplete data records were rated group C. Via statistical models, the MMR for Indonesia for the period 1998 – 2003 was estimated 420 (240–600)/100 000 [9]. Many threshold countries, such as India, Brazil, Egypt, and few southeast countries, among others, belong to groups C–E where at least limited primary data were available. Indonesia may serve as example for these kind of countries regarding data availability.

The estimates of Hogan et al. showed large differences compared to Hill et al. These authors already pointed out their different methods, such as larger database and a systematic search for references resulting in consideration of DHS data, WHO estimates and two subnational verbal autopsies. Their assessment was based on a two–step regression model considering various covariates (total fertility rate, GDP per capita, HIV sero–prevalence, neonatal mortality, age–specific female education, skilled birth attendance) and indicators for 5–year age groups [10].

Lozano et al. apply a concept similar to Hogan, but included more data and observations, such as 60 sibling histories that are roughly comparable with 5–year intervals in IDHS surveys. They included more covariates (age–specific fertility rate, in–facility delivery, total fertility rate, skilled birth attendance, antenatal care coverage, female education by age, HIV prevalence, health system access, neonatal death rate, malnutrition, income) for their linear regression with random effects by country or as the mean function in a Gaussian Process Regression [11].

Kassebaum et al. applied the most extensive study, but for Indonesia these authors used a smaller data set compared to Lozano et al. [11]. Continuing the concept of Lozano et al. they used the Cause of Death Ensemble model (CODEm) to model maternal mortality and considered 9 covariates (age–specific fertility rate, total fertility rate, age–standardized HIV death rate for female individuals aged 15–49 years, neonatal death rate, GDP per person, proportion of deliveries occurring in facilities, proportion of deliveries overseen by skilled birth attendants, coverage of four visits of antenatal care, and malnutrition in children younger than 5 years) [12]. The calculated decline in MMR was comparable for Lozano et al. (ARR 1990–2011: –2.4%) and Kassebaum et al. (ARR 1990–2013: –2.6%).

In contrast to the other model–based MMR estimations Ronsmans et al. carried out a regional case-controlling study with a randomly selected 458 cases and 1.234 controls. This group reported a MMR of 435/100 000 with large differences between rural regions (706/100 000) and urban areas (232/100 000). The authors, however, already discussed some advantages and limitations of their effort to gain retrospective primary data. The quality of delivery documentation may be inaccurate. However, this limitation they considered less relevant in Indonesia, where birth attendants are usually midwives or physicians compared to countries with usually traditional birth attendants. MMR estimates produced by the capture–recapture method used in this evaluation are likely to be less biased than crude death counts regarding missing events. Finally, the applied asset–based classification of the population into wealth quartiles may not overlap with the governmental method the government uses to stratify population cohorts [13].

### Discussion

**Figure 1:**
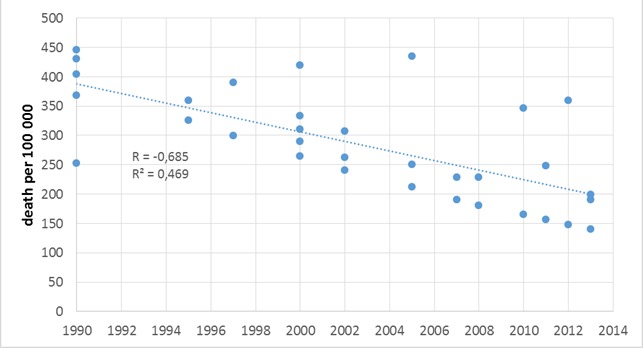
Time course of reported MMR data. Due to the very limited amounts of investigations confidential intervals are not calculated. An overall sufficient trend line can be estimated, but variability of data are remarkable.

In this review we systematically analyzed the available data about maternal mortality in Indonesia as an example to validate the achievements regarding the Millennium Goals of the UN. **Figure 1** shows how different the data of the MMR are.

Taken together the very limited amount of available primary data, their overall data quality, heterogeneity in data acquisition, selection biases, variability in data evaluation and calculation methods as well as lack of sufficient reporting intensively limit evidence based evaluation of the maternal mortality throughout the MDG time frame. Going back to the acquired primary data it became clear that for Indonesia, and very likely for most of developing and threshold countries, reliable estimations of MMR or comparable indicators remains difficult.

Almost all authors use the IDHS–Data for their calculation which has shown to have limitations. Furthermore, due to low frequencies of maternal deaths and the sample size used for DHS surveys standard errors were high for estimates leading to volatile maternal mortality indicators. In addition, the majority of non–sampling errors were also rather under– than overestimated [21,22]. Therefore, reported MMR seem to be not suitable for controlling achievement of MDG targets. Primary data and subsequent maternal mortality models suffer from many restrictions, random and systematic errors resulting in very high uncertainty internals that are most prominent in threshold countries, such as in Southern Asia and Oceania.

However, with exception of one outlier in 2012 the overall correlation coefficient of R = –0.685 (R^2^ = 0.469) seems to support an estimation of the development over time (**Figure 1**). The applicable ARR of the respective data which could be used has been shown in **Table 3**. The ARR obtained by the different data resources is highly variable between 0.6% and 4.9%, but the obtained linear regression of all identified MMR data provides an assumption of an ARR = –2.8% for the whole period of 1990–2014. This regression with an overall reduction of about 50% during the MDG period is in line with the recently published estimates by the UN Maternal Mortality Estimation Inter–Agency Group [16]. Considering these results Indonesia belongs to the vast majority of countries that have been grouped into the medium achievers by the WHO group that made significant progress but did not fully reach initial reduction goals.

**Table 3:** Calculation of annual rate of reduction (ARR) as published in available reports*

Author/Source	MMR				ARR	Years
**1990**	**2008**	**2011**	**2013**
MMEIG	430			190	–3.5%	1990–2013
UN	446			140	–4.9%	1990–2013
Hogan et al.; [10]	253	229			–0.6%	1990–2008
Lozano et al.; [11]	404		248		–2.4%	1990–2011
Kassebaum et al.; [12]	368			199	–2.6%	1990–2013
Overall regression					–2,8%	1990–2013

MMEIG – Maternal Mortality Estimation Group, UN – United Nations

*Overall regression was done using all identified data (compare with **Figure 1**).

Data availability with sufficient coverage of the regions and data quality including standardized reporting formats remain a key issue for a sufficient evaluation of maternal mortality in these countries. Central population statistics with information about births, deaths and causes of death are obligatory data requirements for the improvement of the health care systems. To build up such databases suitable reporting systems, such as web–based solutions or other types of online reporting and telemedicine may provide achievable ways for developing and threshold countries. In addition, capture–recapture sampling has been recommended as reliable technique to obtain MMR estimates for Indonesia and comparable countries [23], but as a survey alternative it also lacks complete coverage of the whole country and maternal mortality cases resulting in high potential sampling errors [16]. We would therefore not consider this method as alternative for the required data quality, especially in the clinically required context. Therefore, the health care policy conclusions drawn by the UN Maternal Mortality Estimation Inter–Agency Group towards clinical efforts to extent achievements of the MDG into a Sustainable Development Goal (SDG) should be broadened with inclusion of efforts to improve health care reporting systems. For example, according to current plans of the “Indonesian Ministry of Foreign Affairs” a central registration system shell be implemented in Indonesia until 2024 [24]. Nevertheless, these data are of considerable relevance for the development of care structures for the improvement of the maternal health but need to be complemented with a basic data set for the delivery process including pre–, peri– and postpartal indicators. Only these types of data would enable detailed conclusions about required actions to achieve SDGs.

Valid data about important cofactors, such as age at delivery of the mothers, number of the exemptions (experience of the obstetric institution), cause of death, etc. are necessary for the improvement of the maternal health and to carry out suitable measures for the medical care development according to the real local/regional/national requirements.
